# Evidence of distinct *RELN* and *TGFB1* genetic associations in familial and non-familial otosclerosis in a British population

**DOI:** 10.1007/s00439-018-1889-9

**Published:** 2018-05-04

**Authors:** Andrew J. Mowat, Michael Crompton, Joanna L. Ziff, Christopher P. Aldren, Jeremy A. Lavy, Shakeel R. Saeed, Sally J. Dawson

**Affiliations:** 1 UCL Ear Institute, University College London, London, WC1X 8EE UK; 2 Department of ENT Surgery, The Princess Margaret Hospital, Windsor, SL4 3SJ UK; 3 Royal National Throat Nose and Ear Hospital, London, WC1X 8EE UK

## Abstract

Otosclerosis is a common form of hearing loss which typically presents in young adults. The disease has a familial, monogenic form and a non-familial form with a more complex aetiology. A previous genome wide association study identified evidence that variants within *RELN* are associated with the condition. Other genes in which an association has been reported include *BMP2, COL1A1, FGF2, PPP2R5B* and *TGFB1*. However, follow up studies have often failed to replicate initial positive results. The aim of this study was to establish if an association exists between eight single nucleotide polymorphisms (SNPs) in these six previously implicated genes and otosclerosis in a British case–control cohort (*n* = 748). Evidence of an association between rs1800472 in *TGFB1* and otosclerosis was found (*p* = 0.034), this association was strongest amongst non-familial cases (*p* = 0.011). No evidence of an association was detected with variants in *COL1A1, FGF2, BMP2*, and *PPP2R5B*. No association between variation in *RELN* and otosclerosis was observed in the whole cohort. However, a significant association (*p* = 0.0057) was detected between one *RELN* SNP (rs39399) and otosclerosis in familial patients. Additionally, we identify expression of one *RELN* transcript in 51 of 81 human stapes tested, clarifying previous conflicting data as to whether *RELN* is expressed in the affected tissue. Our findings strengthen the association of *TGFB1* (rs1800472) with otosclerosis and support a relationship between *RELN* and familial otosclerosis only, which may explain previous variable replications.

## Introduction

Otosclerosis is a common form of progressive, adult hearing loss. The disease is characterised by dystrophy localized to the bone of the otic capsule (Chole and McKenna 2001). A conductive hearing loss develops, as otosclerotic foci invade the stapedio-vestibular joint at the oval window, or the junction of the incus and stapes. Affected individuals may also develop a profound sensorineural loss (Ishai et al. 2016). The only available treatment is two separate surgeries to replace the defective stapes bone with a micro-prosthetic device, which carries the risk of further hearing loss (Mann et al. 1996). The clinical prevalence of the condition has been estimated to be 0.3–0.4% amongst Caucasians (Declau et al. 2001). The age of onset varies from the first to the fifth decade, but most commonly presents in the third (Cawthorne 1955) and this is maintained within families (Chole and McKenna 2001; Morrison 1967).

The disease exists in familial and non-familial forms. The familial form, accounting for between 25–50% of cases, displays an autosomal dominant inheritance pattern with incomplete penetrance (Morrison 1967). Eight different genetic loci have been reported based on genetic mapping in families. However, none of the causal genes within these loci have yet been identified (Ealy and Smith 2010). This is likely to be due to the reduced power of linkage studies in the presence of incomplete penetrance. A more recent study using a whole exome sequencing approach identified multiple individuals with rare mutations in *SERPINF1* in a cohort of familial patients (Ziff et al. 2016). Functional studies suggest these mutations affect expression of an alternatively spliced transcript which is normally highly expressed in stapes bone. Non-familial otosclerosis is considered as a complex disorder which exists as isolated cases with a mixed environmental and genetic aetiology (Ealy and Smith 2010). Whether mutations within the same genes contribute to a common pathology in both familial and non-familial types is currently unclear.

The strongest evidence linking any single gene to the disease came from a genome wide association study (GWAS) in a cohort of patients from Belgium, Holland and France (Schrauwen et al. 2009a). Schrauwen et al. (2009a) performed a case–control discovery experiment utilizing 555,000 single nucleotide polymorphisms (SNPs). The group discovered two highly associated SNPs that were replicated in additional independent populations. The region with the strongest association signal was found on chromosome 7q22.1 (rs39395, *p* = 6.23 × 10^−10^) within the RELN gene. A secondary region of association was found at chromosome 11q13.1 at the *PPP2R5B* locus (rs616322, *p* = 2.20 × 10^−5^). *RELN* codes for the protein reelin, which has a role in neuronal migration. It enables detachment of neurons from radial glial cells (Dulabon et al. 2000). Failure of this migration is associated with neurological and psychiatric disorders, including autism (Fatemi et al. 2005). However, there is no evidence that the bone dysregulation that underlies otosclerosis has any relationship to reelin’s known biological function. Therefore, the association suggested between *RELN* and otosclerosis is a surprising one. Schrauwen et al. (2009a, b) have detected expression of *RELN* in human and mouse stapes, supporting a role for *RELN* in stapes bone (Schrauwen et al. 2009a). A subsequent study could not detect *RELN* in adult human stapes, although the *RELN* protein was present (Csomor et al. 2012).

Subsequent replication of this genetic association between *RELN* and otosclerosis has been inconsistent. The same group who performed the GWAS found supportive evidence when analysing 591 patients from Germany, Italy, Switzerland and Romania (Schrauwen et al. 2010). Similarly, an independent replication using 149 Tunisian cases and 152 controls found evidence of association with three *RELN* SNPs (Khalfallah et al. 2010). However, other studies have failed to replicate the association. A case–control association study excluded any association between the rs39335 SNP and the disease in a southern Italian population of 92 cases and controls (Iossa et al. 2013). Similarly, a study in an Indian population failed to show an association between four separate *RELN* SNPs (rs3914131, rs3914132, rs4641319, and rs10227303), when 170 otosclerosis cases were compared with 170 controls (Priyadarshi et al. 2010). Furthermore, a study in a Hungarian population of 153 cases and 300 controls genotyped thirteen SNPs across six candidate genes (*COL1A1, TGFB1, BMP2, BMP4, AGT, RELN*). Only the association between *TGFB1* and otosclerosis was replicated (Sommen et al. 2014). A number of these studies were underpowered for detection of a *RELN* association. Therefore, tests of such an association in an adequately powered study are required, such as the cohort used here, to ascertain the reliability of the *RELN* association.

Here, we genotyped eight previously associated variants in the following six candidate genes: *RELN, BMP2, FGF2, COL1A1, TGFB1, and PPP2R5B* in a large UK case–control otosclerosis cohort (*n* = 748). In addition, we also investigate the expression of *RELN* transcripts in human stapes and resolve the previous controversies in reelin expression in human stapes.

## Materials and methods

### Patient recruitment

Individuals with a confirmed clinical diagnosis of otosclerosis were recruited from the Royal National Throat Nose and Ear Hospital (London, UK), Princess Margaret Hospital (Windsor), Sunderland Royal Hospital (Sunderland), Freeman Hospital (Newcastle upon Tyne) and Ninewells Hospital (Dundee). The study was approved by the London Bloomsbury NRES Ethics committee (11/LO/0489). The diagnosis of otosclerosis was made on clinical and audiometric examination, and then confirmed during surgery. Patients and controls were recruited by informed consent. Controls had otosclerosis ruled out on the basis of a pure tone audiogram. Blood or saliva samples were obtained for genomic DNA isolation (Oragene^®^ Saliva Kit, DNA Genotek) by standard methods. Patients completed a questionnaire recording family history of otosclerosis and relevant medical history. Female patients answered additional questions recording history of pregnancy, oral contraceptives and hormone replacement therapy. For stratified analysis based on questionnaire data, patients were categorised as familial or non-familial based on reporting an additional first or second-degree family member with a clinical diagnosis of otosclerosis as reported by the proband.

### Genotyping of SNPs

SNPs were genotyped using Applied Biosystems TaqMan Pre-Designed SNP Genotyping assays on an Applied Biosystems 7500 Real Time PCR System according manufacturer’s instructions. Assays IDs were: rs3178250 (*BMP2*), C__27466541_10; rs1800012 (*COL1A1*), C___7477170_30; rs17407577 (*FGF2*), C__34186732_10; rs39335 (*RELN*), C____758635_10; rs39399 (*RELN*), C__2415500_10; rs3914132 (*RELN*), C__7594810_10; rs1800472 (*TGFB1*), C___8708464_20; rs616322 (*PPP2R5B*), C____633165_10.

### Association testing

Tests for genetic association with disease were performed using logistic regression analysis using the SNPstats platform (Sole et al. 2006) under different genetic inheritance models (dominant, recessive and additive), and to investigate covariate interactions with gender and ethnicity. A *p* value of < 0.05 was considered significant. None of the SNPs showed a deviation from Hardy–Weinberg equilibrium in cases or controls. To investigate whether the relationship between the SNPs and otosclerosis was gender-dependent we ran the overall association test using gender as a categorical co-variate. With seven of the eight SNPs, no statistically significant interaction with gender was observed. For rs17407577 in *FGF2* a weak association with gender was observed (*p* = 0.034). In our cohort, 94% of the sample was of white European origin, no interaction was detected when ethnicity was included as a categorical variable. The tests of association were performed separately in white Europeans alone and with participants of all ethnicity and gave similar results.

### Power calculations

Power calculations were performed using the Genetic Power Calculator (http://zzz.bwh.harvard.edu/gpc/) (Purcell et al. 2003). All calculations were performed assuming a disease prevalence of 0.35%, setting the relative risk and disease allele frequency based on previous studies under an additive model (Sommen et al. 2014).

### *RELN* expression in human stapes

Stapes suprastructures were collected from 76 individuals undergoing laser-assisted stapedotomy surgery at the Royal National Throat Nose and Ear Hospital (London, UK). A diagnosis of otosclerosis was confirmed during surgery in all cases. Five additional stapes suprastructures and one whole stapes were obtained from individuals who did not have otosclerosis and who were undergoing surgical procedures during which the stapes were removed. These included surgery due to head trauma, glomus tumour and total petrosectomy. Human stapes were preserved in AllProtect™ Solution (Qiagen) and stored at − 80 °C. Stapes were homogenized in QIAzol Lysis reagent and RNA purified using the RNeasy lipid tissue mini kit (Qiagen) according to the manufacturer’s protocol, including an on column DNase digest. RNA was also extracted from homogenised human MG-63 cells, a human osteosarcoma cell line using the RNeasy mini kit (Qiagen) in accordance with the manufacturer’s protocol. RNA was reverse transcribed into cDNA by Omniscript Reverse Transcriptase Kit (Promega) for RT-qPCR.

Sequence-specific primers were designed to specifically amplify *RELN* transcripts *RELN-201* (Ensembl Transcript ID: ENST00000343529.9) and *RELN-203* (Ensembl Transcript ID: ENST00000428762.5) based on the sites of primers used in previous studies to detect *RELN* expression and with reference to the exon/intron boundaries. Primer sequences: RELN-201F, *5′-*GCAATACAGCGTCAACAACGG-3′; RELN-201R, 5′-GTTTGCGAGTGAGGACGACCT-3′; RELN-203F, 5′-ACCATGTGGAGGTCGTCCTAGTA-3′ and RELN-203R, 5′-CACTCGGTCTTGAGAAGGGCTTT-3′. For all cDNA samples, a GAPDH PCR assay was performed as a control for cDNA integrity and a non-template control for background contamination. MG-63 cDNA was used as a positive control. PCR reactions contained 400 ng cDNA, 0.5 µM each primer, 0.3 mM dNTPs, 2 mM MgCl2, 5 µl of 5× GoTaq PCR buffer and 1.25U GoTaq polymerase (Promega) in a total volume of 20 µl. Thermal cycling was performed under the following reaction conditions: denaturation at 93 °C for 15 s, annealing at optimum temperature for 30 s, extension at 72 °C for 30 s, for up to 40 cycles, with an initial denaturation step of 93 °C for 3 min on an Eppendorf Mastercycler Gradient machine. The resulting DNA bands were visualised by agarose gel electrophoresis.

## Results and discussion

Genome wide and candidate gene association studies represent powerful genetic tools. However, such studies can generate false positive associations. Replication in independent populations is, therefore, vital for discriminating true associations from false positives. An important limitation to the replication of genetic associations within otosclerosis has been the lack of well characterised patient cohorts of sufficient size for replication studies. We have genotyped eight previously implicated SNPs in a novel British cohort of 374 patients and 374 controls. Association with disease was tested by logistic regression analysis under various genetic models. Power calculations (Table 1) estimated that the study had good power to detect most SNP effects (between 70 and 99.9% power for six SNPs) with only *TGFB1* and *COL1A1* having limited power to detect an effect, at 48.9 and 45.0%, respectively.

**Table 1:** Power calculation for each SNP within the British case–control cohort

SNP	Gene	DAF	OR	Power (%)
rs39399	*RELN*	0.49	1.45	99.9
rs3914132	*RELN*	0.78	1.54	99.7
rs39335	*RELN*	0.15	1.35	81.8
rs3178250	*BMP2*	0.76	2.03	99.9
rs1800012	*COL1A1*	0.82	1.55	45.0
rs17407577	*FGF2*	0.03	2.00	76.1
rs1800472	*TGFB1*	0.97	2.33	48.9
rs616322	*PPP2R5B*	0.08	1.46	70.0

Power calculations were performed based on previously reported data (Chen et al. 2002; Sommen et al. 2014)

*DAF* disease allele frequency, *OR* odds ratio

Results for tests of genetic association with otosclerosis in all cases and controls are shown in Table 2. Only the rs1800472 variant within the *TGFB1* gene (*p* = 0.021) was found to be significantly associated with otosclerosis. This SNP was selected for a case–control association study due to several genetic indications of involvement with the disease. Thys et al. (2009), performed a case–control association study in a Belgian-Dutch cohort which gave significant results (*p* = 0.0044) (Thys et al. 2009). Within the same study, the association was replicated in a French population, with even greater significance (*p* = 0.00019). Our study represents the third subsequent case–control association study which has confirmed the association (Khalfallah et al. 2010; Sommen et al. 2014). The evidence for the association is increased further by the absence of any conflicting studies which have failed to demonstrate an association. Furthermore, much of this supportive evidence has come from studies which are underpowered to detect the SNP’s effect. All studies have found the disease to be associated with the major allele suggesting the variant is protective. The SNP is relatively rare with a minor allele frequency of 0.024 and 0.045 in cases and controls respectively.

**Table 2:** Association testing of the eight SNPs in the full case–control cohort (*n* = 748)

SNP	Gene	*p*	OR (95% CI)
rs39399	*RELN*	0.32	1.17 (0.86–1.60)
rs3914132	*RELN*	0.33	1.16 (0.86–1.58)
rs39335	*RELN*	0.38	1.16 (0.84–1.59)
rs3178250	*BMP2*	0.65	0.93 (0.70–1.25)
rs1800012	*COL1A1*	0.21	1.13 (0.84–1.53)
rs17407577	*FGF2*	0.45	0.85 (0.55–1.30)
rs1800472	*TGFB1*	**0.021**	**0.51 (0.28–0.91)**
rs616322	*PPP2R5B*	0.38	0.86 (0.62–1.20)

Significant values are indicated in bold. *p* values are shown for a test of association under the dominant model. All odds ratios quoted are for the minor allele

The rs1800472 SNP is a missense variant that causes a threonine to isoleucine substitution at amino acid position 263 in TGFB1, this change has been shown to effect activity of the protein (Thys et al. 2009). Analysis of the T263I substitution with a luciferase reporter assay showed that the protective variant of TGFB1 is more active than the major allele. The reason why a more active version of the protein might be protective against otosclerosis is unclear (Thys et al. 2007). Our results, combined with previous similar association studies, suggest that the mutant form of the protein is protective against otosclerosis.

The lack of association between any of the three *RELN* SNPs and otosclerosis is surprising given the high power of the study to detect an effect for these SNPs. Otosclerosis has both familial and non-familial forms and approximately 44% of our patient cohort reported other family members with a diagnosis of otosclerosis. Since it is possible that there are distinct genetic susceptibilities involved in the two forms of the disease we performed an analysis when stratifying the patient cohort by familial or non-familial disease, see Tables 3 and 4 respectively. In familial cases a strong association was detected between rs39399 and otosclerosis (*p* = 0.0057, Table 3) that was not present in non-familial cases (Table 4). As with the association seen in *TGFB1*, it is the major allele that is associated with disease, replicating the direction of association detected in the original GWAS. Our results suggest that an association exists between rs39399 and the familial form of otosclerosis, which requires further investigation in familial cohorts. No association between rs1800472 in *TGFB1* and disease was detected in familial cases (Table 3), however, in non-familial cases an association was again detected this time with a lower *p* value (*p* = 0.015, Table 4) and greater odds ratio than was detected in the combined otosclerosis cases, suggesting that this SNP may be playing a role in the non-familial form of the disease only.

**Table 3:** Association testing in familial otosclerosis cases (*n* = 160) and controls (*n* = 374)

SNP	Gene	*p*	OR (95% CI)
rs39399	*RELN*	**0.0057**	**1.73 (1.18–2.54)**
rs3914132	*RELN*	0.17	1.33 (0.89–1.99)
rs39335	*RELN*	0.30	1.25 (0.82–1.90)
rs3178250	*BMP2*	0.54	0.89 (0.61–1.30)
rs1800012	*COL1A1*	0.30	1.25 (0.82–1.90)
rs17407577	*FGF2*	0.84	0.96 (0.65–1.41)
rs1800472	*TGFB1*	0.28	1.48 (0.71–3.08)
rs616322	*PPP2R5B*	0.25	0.78 (0.52–1.18)

Significant values are indicated in bold. *p* values are shown for a test of association under the dominant model. All odds ratios quoted are for the minor allele

**Table 4:** Association testing in non-familial otosclerosis cases (*n* = 206) and controls (*n* = 374)

SNP	Gene	*p*	OR (95% CI)
rs39399	*RELN*	0.40	0.85 (0.58–1.24)
rs3914132	*RELN*	0.55	1.00 (0.70–1.43)
rs39335	*RELN*	0.52	1.04 (0.71–1.51)
rs3178250	*BMP2*	0.92	0.98 (0.69–1.39)
rs1800012	*COL1A1*	0.24	1.24 (0.86–1.78)
rs17407577	*FGF2*	0.90	0.99 (0.59–1.67)
rs1800472	*TGFB1*	**0.015**	**0.40 (0.18–0.88)**
rs616322	*PPP2R5B*	0.71	0.89 (0.60–1.33)

Significant values are indicated in bold. *p* values are shown for a test of association under the dominant model. All odds ratios quoted are for the minor allele

It has been well documented that some women with otosclerosis associate the onset or worsening of hearing loss with pregnancy and it has been postulated that this may be due to the influence of oestrogen (Torsiglieri et al. 1990). Approximately 40% of the females in our cohort who had experienced a pregnancy self-reported a worsening of hearing with pregnancy in questionnaire data. To explore whether these females represent a distinct genetic aetiology from the main cohort we tested association with disease in females stratified by this self-reported association with pregnancy (Table 5).

**Table 5:** Association testing in women stratified by reported hearing loss associated with pregnancy

SNP	Gene	No pregnancies *p* value (*n* = 55)	Pregnant change *p* value (*n* = 65)	Pregnant no change *p* value (*n* = 99)
rs39399	*RELN*	0.28	0.95	0.094
rs3914132	*RELN*	0.89	0.22	**0.0073** ^**a**^
rs39335	*RELN*	0.75	0.76	**0.038** ^**a**^
rs3178250	*BMP2*	0.86	0.63	0.70
rs1800012	*COL1A1*	0.75	0.60	0.47
rs17407577	*FGF2*	0.19	0.60	0.39
rs1800472	*TGFB1*	0.36^a^	0.67^a^	0.17^a^
rs616322	*PPP2R5B*	0.93	0.21^a^	0.20

Significant values are indicated in bold. *p* values are shown for a test of association under the dominant model unless otherwise indicated

^a^Recessive model

No association with otosclerosis was detected with any of the eight SNPs in those women who reported worsening hearing in pregnancy or those who had not been pregnant. A significant relationship was seen with the *RELN* SNPs, rs3914132 (*p* = 0.0095) and rs39335 (*p* = 0.038), in those women who did not report a deterioration in their hearing when compared with controls. These data should be treated with caution as the stratified sample sizes are small and, therefore, more prone to error. If substantiated by replication in other cohorts our results suggest that *RELN* may play a role in the pathogenesis of otosclerosis, but only in specific subtypes. Indeed our data when taken together suggest that otosclerosis has a complex pathophysiology, with non-familial and familial cases having different underlying genetic mechanisms, each involving separate genetic pathways.

Previous association studies with *RELN* and otosclerosis have not discriminated between cases with familial and non-familial disease. If our finding of *RELN* association with familial disease only is substantiated it is possible that the proportion of patients with familial disease in cohorts used in previous studies may have influenced the ability to detect this association and account for variable replication between studies. For example, variant rs3914132 has now been included in five follow up case–control association studies following its identification in the original GWAS. Two of those studies have replicated the initial association (Khalfallah et al. 2010; Schrauwen et al. 2009a). However, two others have failed to display the replication (Priyadarshi et al. 2010; Sommen et al. 2014). The results also suggest a hypothesis that the otosclerosis phenotype maybe reached, through an oestrogen dependent or independent pathway. Those cases which are oestrogen dependent, progress disproportionately during pregnancy. Our data suggest that *RELN* may be exclusively involved in the oestrogen independent mechanism. If so, the results of previous case–control association studies may have been determined by the make-up of each cohort which may have been influenced by differences in recruitment pipelines.

**Fig. 1:**
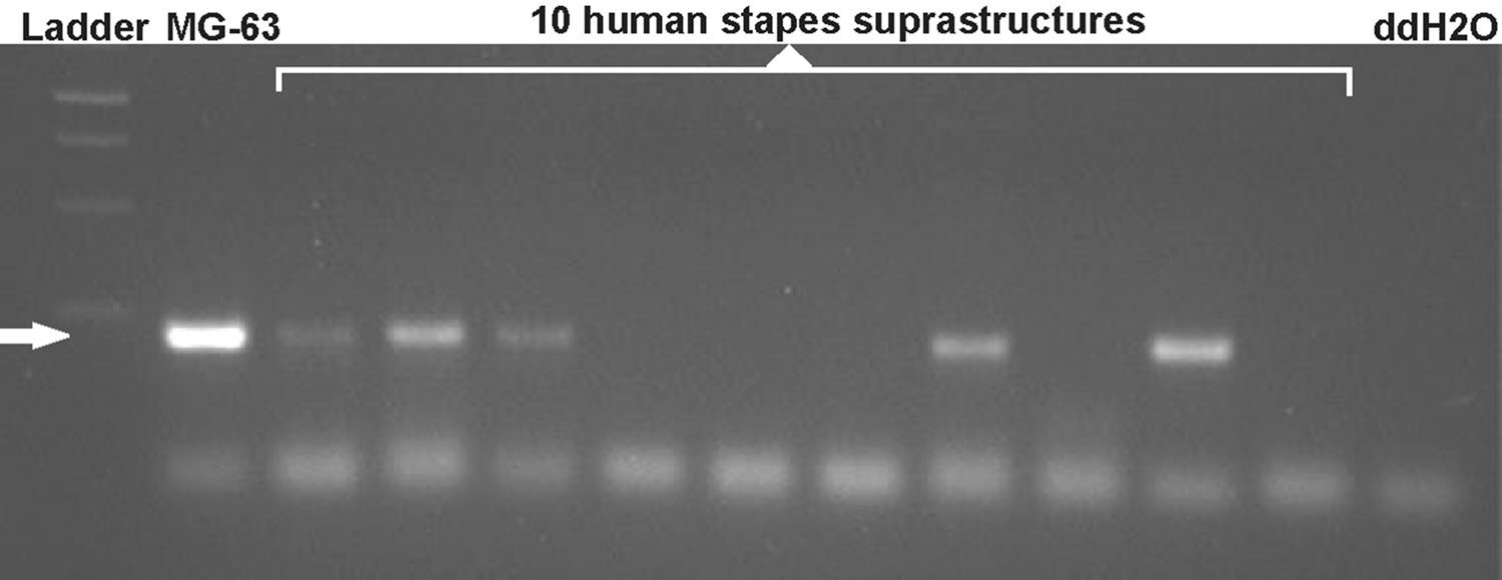
A 2% agarose gel showing *RELN-201* RT-PCR amplicons detected in human stapes cDNA. *RELN-201* was detected in MG-63 cells and five out of ten human stapes suprastructures shown (a representative sample of the cohort), illustrated by a 209 bp fragment (arrowed)

Another factor in considering whether *RELN* is involved in the pathogenesis of otosclerosis has been the lack of an obvious role for the reelin protein in stapes bone and the uncertainty as to whether it is expressed in human stapes. Two previous studies reported conflicting results in detecting *RELN* transcripts in stapes bone (Csomor et al. 2012; Schrauwen et al. 2009b). The current release of human genome assembly (Ensembl: GRCh38.p10, accessed November 2017) shows that the predicted major transcripts of *RELN* diverge in the use of different microexons 64 (no exon, 4 bp exon or 6 bp exon) at the 3′ end of the transcript. Examination of the primers used in previous studies suggested that the assays used may be detecting different transcripts (*RELN-201* in Schrauwen et al. 2009a) and (*RELN-203* in Csomor et al. 2012), which might account for the different results. We therefore designed assays to detect both *RELN-201* and *RELN-203* in human stapes. The results (Table 6; Fig. 1) show that the *RELN-201* transcript is detected in the majority of stapes suprastructures removed from both otosclerosis patients and controls. However, the *RELN-203* transcript was not detected in any human stapes, but was detected in the positive control, cDNA from the MG-63 cell line. Our results clarify the expression of *RELN* in human stapes and are similar to those reported by Schrauwen et al. (2009a, b) suggesting that reelin is expressed in the majority but not all of human stapes tested (Schrauwen et al. 2009a).

**Table 6:** *RELN* expression in human stapes

Sample (*n*)	No. (%) of samples in which transcript is detected		
	*GAPDH*	*RELN-201*	*RELN-203*
MG-63 cells (1)	1 (100%)	1 (100%)	1 (100%)
Female stapes suprastructures (52)	52 (100%)	32 (62%)	0 (0%)
Male stapes suprastructures (19)	19 (100%)	11 (58%)	0 (0%)
Control suprastructures (5)	5 (100%)	4 (80%)	0 (0%)
Total stapes suprastructures (76)	76 (100%)	47 (62%)	0 (0%)

No statistically significant association was seen between four SNPs in *COL1A1, FGF2, PPP2R5B, or BMP2* and otosclerosis across all comparisons. This provides further evidence against the involvement of these genes in otosclerosis disease pathogenesis. For some of the SNPs in question, such as rs1800012 in *COL1A1*, this is the third consecutive case–control association study (Khalfallah et al. 2010; Sommen et al. 2014) in which an association has failed to replicate the initial positive association (Chen et al. 2002). However, due to the low odds ratio reported for this association this study has poor power to detect such a weak effect. For other variants the reliability of the negative association is increased by the power of this study. For example, no effect was detected for rs3178250 in *BMP2* despite the study having a 99.9% power to detect it (Table 1).

In conclusion, we detected an association between a functional variant in *TGFB1* and clinically confirmed otosclerosis in a British population. This replicates two previous studies which have found the same association in the same direction. Further, we detect an association between different SNPs within *RELN* and familial otosclerosis and in women whose hearing loss does not progress during pregnancy. A previously described association between four other genes (*COL1A1, BMP2, PPP2R5B, and FGF2*) was not replicated, providing further negative evidence for any involvement of these genes in the pathogenesis of the condition.
